# Reach and effectiveness outcomes of the “I’m Ready” HIV self-testing program in Black communities in Canada

**DOI:** 10.17269/s41997-026-01183-0

**Published:** 2026-03-23

**Authors:** Wale Ajiboye, Jason Lo Hog Tian, Wangari Tharao, Maureen Owino, Lena Soje, Kristin McBain, Amy Ly, Reena Anthonyraj, David Este, Mary Ndung’u, Roger Antabe, Notisha Massaquoi, Lawrence Mbuagbaw, LaRon Nelson, Sean Rourke

**Affiliations:** 1 MAP Center for Urban Health Solution, Unity Health Toronto, Toronto, ON Canada; 2 Women’s Health in Women’s Hands Community Health Center, Toronto, ON Canada; 3 Faculty of Environmental Studies, York University, Toronto, ON Canada; 4 Women’s College Hospital, Toronto, ON Canada; 5 Department of Health and Society, University of Toronto, Scarborough, ON Canada; 6 Dalla Lana School of Public Health, University of Toronto, Toronto, ON Canada; 7 School of Nursing, Yale University, New Haven, Connecticut, ON Canada; 8 Department of Psychiatry, University of Toronto, Toronto, ON Canada; 9 Department of Health Research Methods, Evidence, and Impact (HEI), McMaster University, Hamilton, ON Canada; 10 Faculty of Social Works, University of Calgary, Calgary, AB Canada

**Keywords:** HIV self-testing, Black people in Canada, “I’m Ready” program, Implementation outcomes, Autotest du VIH, Personnes noires au Canada, Programme I’m Ready

## Abstract

**Objectives:**

HIV self-testing is a strategy to scale up HIV testing for communities that face significant barriers to facility-based testing services. The I’m Ready program—an integrated technology solution—is an implementation strategy designed to promote the uptake and use of HIV self-testing kits in Canada. The objective of this study is to examine the reach and effectiveness of the I’m Ready program among Black people in Canada.

**Method:**

We performed a secondary analysis of data for Black people who participated in the I’m Ready implementation study from June 2021 to Dec 2023. We defined reach as the number and percentage of persons with indications for HIV testing who accessed the intervention and effectiveness as the number and percentage of those who ordered test kits and reported test results through the app. Participants’ characteristics and variables of interest were summarized using descriptive statistics.

**Results:**

Overall, 3082 (22%—3082/14,071) Black participants accessed the intervention between June 2021 and December 2023. The intervention reached a majority (86%—1846/2141) of Black people with indications for HIV testing who were either first-time testers (38%—824/2190) or had not tested in the previous 1 year (33%—712/2190). Overall, 29% (894/3082) ordered test kits through the app and 31% (274/894) of those who ordered test kits reported their test results. The majority (72%—240/333) of the test results were negative, 26% (87/333) were invalid, and 2% (6/333) of the results reported were positive.

**Conclusion:**

The I’m Ready program reached Black people with indications for HIV testing and facilitated the uptake of HIV self-testing. It could be a potential way to increase HIV testing in Black communities in Canada.

## Résumé

**Objectifs:**

L’autotest du VIH est une méthode servant à généraliser le dépistage du VIH dans les communautés où le dépistage au point de service est limité. Le programme I’m Ready, une solution technologique intégrée, est une stratégie de mise en œuvre qui vise à favoriser l’adoption et l’utilisation de la trousse d’autotest du VIH au Canada. Nous avons étudié la portée et l’efficacité du programme I’m Ready chez les personnes noires au Canada.

**Méthode:**

Nous avons effectué une analyse secondaire des données des personnes noires ayant participé à l’étude de mise en œuvre du programme I’m Ready entre juin 2021 et décembre 2023. La portée a été définie comme étant le nombre et le pourcentage de personnes chez qui le dépistage du VIH était indiqué qui ont eu recours à l’intervention, et l’efficacité a été définie comme étant le nombre et le pourcentage de personnes ayant commandé la trousse d’autotest et déclaré leurs résultats au moyen de l’appli. Les caractéristiques des personnes participantes et les variables d’intérêt ont été résumées à l’aide de statistiques descriptives.

**Résultats:**

Dans l’ensemble, 3082 (22%—3082/14071) personnes noires ont eu recours à l’intervention entre juin 2021 et décembre 2023. L’intervention a joint la majorité (86%—1846/2141) des personnes noires chez qui le dépistage du VIH était indiqué, qui soit se faisaient tester pour la première fois (38%—824/2190), soit n’avaient pas été testées depuis 1 an (33%—712/2190). Globalement, 29% (894/3082) ont commandé la trousse d’autotest au moyen de l’appli, et 31% (274/894) de ces personnes ont déclaré leurs résultats. La majorité (72%—240/333) des résultats étaient négatifs, 26% (87/333) étaient invalides, et 2% (6/333) étaient positifs.

**Conclusion:**

Le programme I’m Ready a joint les personnes noires chez qui le dépistage du VIH était indiqué et a facilité le recours à l’autotest du VIH. Ce pourrait être un moyen d’accroître le dépistage du VIH dans les communautés noires du Canada.

## Introduction

New HIV infections are on the rise in Canada. In 2023, the national rate of reported newly diagnosed HIV cases was 6.1 per 100,000 population, an increase from 4.7 per 100,000 population in 2022 (PHAC, 2024). Among key populations impacted by HIV in Canada, Black people bear a disproportionate burden of new HIV infections every year. For example, in 2023, for cases where race and/or ethnicity was reported, Black people accounted for 29% of new infections, despite only representing 4.5% of the Canadian population (PHAC, 2024). In addition, research evidence shows that Black people in Canada are less likely than any other racialized group to be aware of their increased risk of acquiring HIV or be aware of their HIV status (Antabe et al., 2021). The reasons for this gap are multidimensional and interconnected, and include individual, structural, and systemic level barriers that impede Black people’s access and utilization of facility-based HIV testing services (Antabe et al., 2021; Djiadeu et al., 2020; Etowa et al., 2022; Konkor et al., 2022). To achieve the UNAIDS target of reaching 95% of undiagnosed by 2030, the World Health Organization (WHO) recommends the use of HIV self-testing as a safe, accurate, and effective way to reach individuals who might not otherwise test, including key populations and those experiencing significant barriers to facility-based testing (WHO, 2023). The Public Health Agency of Canada (PHAC) has also identified reaching the undiagnosed as the first and important strategy to end HIV as a public health threat by 2030 (PHAC, 2018). Therefore, effective implementation of HIV self-testing (HIVST) in Black communities could be an important step toward ending HIV in Canada.

There is strong evidence that demand and acceptability for HIV self-testing are high (Pant Pai et al., 2013; Witzel et al., 2020). Self-testing allows individuals to test themselves in a private and convenient setting, potentially overcoming barriers like stigma, limited access to healthcare facilities, and other barriers associated with traditional facility-based testing (Figueroa et al., 2015; Laprise & Bolster-Foucault, 2021). In addition, studies have shown that self-testing is preferred over certain in-person testing methods due to its convenience and confidentiality, particularly among marginalized and underserved groups (Hoyos et al., 2021; Saul & Gursul, 2021; Zhang et al., 2022). Moreover, after the introduction of HIVST as a testing option by WHO, various strategies have been developed, tested, and implemented to promote access and uptake (Ma et al., 2022; Rivera et al., 2021). In high-income countries, strategies to promote access and uptake vary in design, target population, and outcomes. Community-based strategies include distribution of HIVST kits through community agencies like sexual health clinics (Martínez-Riveros et al., 2024), or during community-based events and use of social network-based approach — peer distribution (Frye et al., 2021; Kwan et al., 2023; Rael et al., 2022). Use of digital technologies to promote access and uptake of HIVST is the most frequent used strategy and includes web-based ordering of test kits (Bell et al., 2021; Chan et al., 2021; O'Byrne et al., 2023; Rodger et al., 2022), use of mobile app (Sullivan et al., 2022; Wray et al., 2020), and social media platforms (Facebook, Instagram, dating app) (MacGowan et al., 2024; Young et al., 2022). Target populations for HIVST include men who identify as gay or bisexual and other men who have sex with men (gbMSM), Black and Latino communities, female sex workers, and transgender people (Bell et al., 2021; Chan et al., 2021; Frye et al., 2021; Kwan et al., 2023; Ma et al., 2022; MacGowan et al., 2024; Martínez-Riveros et al., 2024; O'Byrne et al., 2023; Rael et al., 2022; Rivera et al., 2021; Rodger et al., 2022; Sullivan et al., 2022; Wray et al., 2020; Young et al., 2022). The most frequently reported primary outcomes include proportion of first-time testers reached and uptake of HIVST kits (Bell et al., 2021; Chan et al., 2021; Frye et al., 2021; Kwan et al., 2023; Ma et al., 2022; MacGowan et al., 2024; Martínez-Riveros et al., 2024; O'Byrne et al., 2023; Rael et al., 2022; Rivera et al., 2021; Rodger et al., 2022; Sullivan et al., 2022; Wray et al., 2020; Young et al., 2022).

In 2020, Health Canada approved the first HIV self-testing kit — the finger-stick blood-based HIV Self-Test kit (BioLytical Galli et al., 2021; Laboratories, 2026), presenting a potential opportunity to overcome the significant barriers to traditional facility-based HIV testing faced by many communities in Canada. To promote access and uptake of the HIVST kits, a national community-based implementation program — the “I’m Ready*”* national HIV self-testing program — was developed and implemented from 2021 to 2023. This was the first attempt at implementing HIV self-testing nationally in Canada; therefore, understanding the implementation outcomes of the “I’m Ready” intervention is crucial for informing public health strategies and ensuring equitable access to HIV testing and care for marginalized communities, especially communities that bear a disproportionate burden of HIV infection. Findings from this study will also provide valuable insights into the program’s reach and effectiveness, supporting the ongoing development and scaling up of the intervention.

The objective of this article is to describe the implementation outcomes of the “I’m Ready” program in Black communities with a focus on reach and effectiveness outcomes. The goal of the evaluation is to answer the following questions: (1) Did the intervention reach the target population in Black communities in Canada? (2) Was the intervention effective in promoting uptake of HIV self-testing among the target population in Black communities?

## Methods

### Study design

The “I’m Ready” program is an observational implementation study of HIV self-testing in Canada. Data for study participants who identified as Black in the “I’m Ready” study were analyzed to determine the implementation outcomes in Black communities.

### Description of the intervention—“I’m Ready” HIV self-testing program

The “I’m Ready” HIV self-testing program is a community-based intervention with three distinct yet integrated components—a community mobilization strategy (which comprises steering committees and social mobilization through various social media platforms), an online technology solution (mobile app and website), and a virtual telehealth option (with peer navigators). The steering committees provided leadership for community mobilization, promotion of the HIV self-testing platforms, and engagement of different stakeholders for participation in the HIV self-testing program. The online technology solution comprises an integrated platform of a mobile app, a public website, and a virtual telehealth. Through this platform, anyone (age 18 years and above) interested in HIV testing could order self-testing kits (one to three kits) to be mailed to any address of their choice, receive support to perform the test, and information for linkage to care. The virtual telehealth is the component that supports testing and linkage to care for testers. Through the virtual telehealth, trained peer navigators provide support for participants for linkage to HIV prevention services (for those testing negative) or HIV treatment (for those testing positive).

### Implementation of the “I’m Ready” intervention

Ethics approval for the study was received from the Unity Health Toronto Research Ethics Board (REB#: 20–283) and the study was conducted between June 2021 and December 2023. To qualify for the “I’m Ready” study, participants needed to be resident in Canada, able to provide online consent in either English or French, be at least 18 years old, have a negative or unknown HIV status, and possess a cellular phone. Individuals who were aware of their HIV-positive status were excluded from the study. Prospective participants were recruited through multiple channels, including community referral (REB-approved advertisements, word of mouth) and public campaigns (including social media and project websites), as well as internet searches. Study participants could download the mobile app from the Apple App Store (for iOS users) and the Google Play Store (Android users). After providing informed written consent in the app, participants would fill in a pre-test survey prior to ordering HIV self-test kits. Participants could order the kits to be delivered to a preferred address or to a nearby participating AIDS Service Organization. After receiving their kit, participants could either perform unassisted self-testing or request for assisted self-testing through the app. Once self-testing is completed, participants had the option to fill in their self-test result in the app. Depending on what the participant filled in for their test result (positive, negative, invalid), further tailored information was given regarding appropriate follow-up and linkage to care (e.g., confirmatory HIV testing, HIV prevention and pre-exposure prophylaxis, and local community organizations).

All data collected from the app was stored on private and secure servers in Canada with the Amazon Web Services (AWS) secure cloud. Data was anonymized, password protected, and accessible only by approved team members at Unity Health Toronto.

### Measures

Using the Reach, Effectiveness, Adoption, Implementation, and Maintenance (RE-AIM) framework, we evaluated the reach and effectiveness outcomes of the “I’m Ready” intervention in Black communities to provide the most valuable insight into the impact of the program. RE-AIM is a commonly used framework to guide the planning and evaluation of programs. The RE-AIM framework is a commonly used tool to improve the adoption and sustenance of implementation of evidence-based interventions in a wide range of health, public health, educational, community, and other settings (Gaglio et al., 2013; Harden et al., 2018).

We assessed “Reach” of the intervention as the number and percentage of the target population — those with indication for HIV testing — who participated in the intervention. We defined target population as those with indications for HIV testing. Indications for HIV testing were based on sexual risk behavior that could lead to HIV infection (PHAC, 2017). In addition, to expand our understanding of the target population reached, we analyzed the data to determine the number and percentage of first-time testers among the target populations, and the locations (provinces and community sizes) of the target population reached.

We accessed “Effectiveness” of the intervention as the absolute number and percentage of participants who ordered test kits through the app, and the number and percentage of those who reported their test results. To expand our understanding of the effectiveness of the “I’m Ready” program, we analyzed the demographic characteristics of those who ordered test kits and those who reported their test results through the app.

### Statistical analysis

A sexual risk indication was determined based on two sexual behavior questions asking about condomless sex in the past 3 months and the number of sexual partners of unknown HIV status in the last 12 months. Participants who did not have condomless sex in the past 3 months and no condomless sex with partners of unknown HIV status in the past 12 months had “low” sexual behavior indicators. Participants who had condomless sex in the past 3 months and condomless sex with at least one partner of unknown HIV status in the past 12 months had “high” sexual behavior indicators. Participants who used injection drugs were also classified as having “high” behavior indicators.

Participant characteristics and key variables of interest were summarized using descriptive statistics. Absolute numbers and corresponding percentages were calculated to describe both reach and effectiveness measures. Percentages were rounded up to whole numbers. All analyses were conducted using IBM SPSS Statistics version 24.

## Results

### Demographic, location, and behavioral characteristics

Overall, 14,071 participants accessed the intervention of whom 3082 (22%) participants identified as Black. Among participants who identified as Black, the majority (86%—1566/3082) were 18–24 years old and many indicated being students (30%—665/2214) (see Table 1). About one in five participants identified as gay, bisexual, or queer, and one in two participants identified as cis-male. The majority (54%—1186/2198) of participants reported having more than a high school education (college or university), and 28% (622/2214) were in full-time employment. The majority (71%—1536/2240) of participants were first-time testers (38%—824/2240) or had not tested in the last 1 year (33%—712/2240). Participants accessed the intervention from all provinces and territories in Canada, and in all various community sizes (see Table 2). The majority (54%—1203/2203) who accessed the intervention live in medium size (30k–99k population) and small size (less than 29k population) communities.

**Table 1:** Demographic characteristics of participants

	***N* (%)**
Age (*n* = 3082)	
18–24	1566 (51)
25–34	1065 (35)
35–44	374 (12)
45–54	60 (2)
55+	17 (1)
Gender (*n* = 3010)	
Cis-man	1596 (53)
Cis-woman	1298 (43)
Non-binary	26 (1)
Trans man	27 (1)
Trans woman	12 (0)
Genderqueer	19 (1)
Self-described gender	32 (1)
Sexual orientation (*n* = 2586)	
Straight	1232 (48)
Gay	183 (7)
Lesbian	30 (1)
Bisexual	252 (10)
Asexual	295 (11)
Heteroflexible	163 (6)
Pansexual	61 (2)
Questioning	58 (2)
Queer	47 (2)
Self-describe sexuality	265 (10)
Education (*n* = 2198)	
> High school	1186 (54)
High school	695 (32)
< High school	317 (14)
Employment (*n* = 2214)	
Full-time	622 (28)
Part-time	229 (10)
Self-employed	348 (16)
Not working/benefits	350 (16)
Student	665 (30)

**Table 2:** Location, testing behavior, and sexual risk characteristics of participants

Characteristic	*N* (%)
Locations	
Province (*n* = 3082)	
Ontario	562 (18)
British Columbia	479 (16)
Prairies	858 (28)
Quebec	296 (10)
Atlantic	416 (14)
Territories	471 (15)
Community size (*n* = 1303)	
Very large urban (> 200k)	678 (31)
Large urban (100k–199k)	322 (15)
Medium (30k–99k)	303 (14)
Small (1k–29k)	900 (41)
Testing history (*n* = 2240)	
First time tester	824 (38)
More than a year ago	712 (33)
3–12 months ago	356 (16)
In the past 3 months	298 (14)
Sexual behavior risk (*n* = 2141)	
Low risk	295 (14)
High risk	1846 (86)

### Reach of the intervention

Overall, 86% (1846/2141) of participants who accessed the intervention had indications for HIV testing (see Table 2). Among these participants, 35% (635/1807) were first-time testers and 32% (584/1807) had not tested in the last 1 year (see Table 3). Participants with indications for HIV testing accessed the intervention from all provinces and territories, and from different community sizes (see Table 3).

**Table 3:** Demographics of participants with indications for HIV testing

	*n*	%
Age (*n* = 1846)		
18–24	881	48%
25–34	678	37%
35–44	240	13%
45–54	39	2%
55+	8	0%
Province (*n* = 1846)		
Ontario	401	22%
British Columbia	273	15%
Prairies	474	26%
Quebec	209	11%
Atlantic	249	13%
Territories	240	13%
Location size (*n* = 1345)		
Very large urban (> 200k)	482	36%
Large urban (100k–199k)	215	16%
Medium (30k–99k)	184	14%
Small (< 29k)	464	35%
Testing history (*n* = 1807)		
Tested before	1172	65%
First time tester	635	35%
Last test for tested before (*n* = 1127)		
In the past 3 months	241	21%
3–12 months ago	302	27%
More than a year ago	584	52%

### Effectiveness of the intervention

Overall, 29% (894/3082) ordered test kits through the mobile app and one in three (31%—274/894) of those who ordered test kits reported their test results (see Table 4). Among those who reported their test results, 61 participants reported test results more than once during the study period. Overall, 333 test results were submitted through the app. The majority 72% (240/333) of the results submitted were negative, 26% (87/333) were invalid, and five participants reported six positive results. The positivity rate is 1.8% (5/274) based on the number of participants who reported test results. The majority (83%—703/894) of those who ordered test kits had indications for HIV testing, had tested before (70%—629/869), and were within the 18–34 years age category (73%—650/894). The majority of those who reported their test results had indications for HIV testing (84%—222/263), had tested before (79%—207/263), and were 18–34 years old (70%—190/274) (see Table 5).

**Table 4:** Effectiveness outcomes for I’m Ready program among Black people in Canada

Indicators	*N *(%)
Participants who ordered test kits through the app	894/3082 (29%)
Participants who reported test result	274/894** (31%)
Total number of results reported	333
Outcome of test results	
- Number of positive results reported	6/333*** (2%)
- Number of negative results reported	240/333 (72%)
- Number of invalid results reported	87/333 (26%)
Positivity rate	5/274 = 1.8%

**61 people reported more than once during the study period

***5 participants reported positive results with one participants reporting positive results twice

**Table 5:** Demographics of participants who ordered test kits and reported test results

Ordered kit			Test submitted		
	*n*	%		*N*	%
Age (*n* = 894)			Age (*n* = 274)		
18–24	251	28%	18–24	65	24%
25–34	399	45%	25–34	125	46%
35–44	195	22%	35–44	67	24%
45–54	38	4%	45–54	14	5%
55+	11	1%	55 +	3	1%
Province (*n* = 894)			Province (*n* = 274)		
Ontario	391	44%	Ontario	140	51%
British Columbia	71	8%	British Columbia	16	6%
Prairies	148	16%	Prairies	34	12%
Quebec	185	21%	Quebec	63	23%
Atlantic	73	8%	Atlantic	20	7%
Territories	26	3%	Territories	1	1%
Gender (*n* = 878)			Gender (*n* = 272)		
Man	480	55%	Man	143	53%
Other	40	4%	Other	13	4%
Woman	358	41%	Woman	116	43%
Sexual Orientation (n = 820)			Sexual Orientation (n = 265)		
Gay/lesbian	97	12%	Gay/lesbian	36	14%
Heterosexual	498	61%	Heterosexual	165	62%
Bisexual	92	11%	Bisexual	23	9%
Other	133	16%	Other	41	15%
Education (*n* = 849)			Education (*n* = 267)		
< High school	52	6%	< High school	8	3%
High school	192	23%	High school	51	19%
> High school	605	71%	> High school	208	78%
Employment (*n* = 840)			Employment (*n* = 265)		
Full-time	376	45%	Full-time	123	46%
Part-time	112	13%	Part-time	36	14%
Self-employed	72	9%	Self-employed	11	4%
Not working/benefits	100	12%	Not working/benefits	30	11%
Student	180	21%	Student	65	25%
Location size (*n* = 721)			Location size (*n* = 228)		
Very large urban (> 200k)	458	63%	Very large urban (> 200k)	159	70%
Large urban (100k–199k)	123	17%	Large urban (100k–199k)	41	18%
Medium (30k–99k)	70	10%	Medium (30k–99k)	20	8%
Small (< 29k)	70	10%	Small (< 29k)	8	4%
Sexual behavior risk (*n* = 844)			Sexual behavior risk (*n* = 263)		
Low risk	141	17%	Low risk	41	16%
High risk	703	83%	High risk	222	84%
Testing history (*n* = 856)			Testing history (*n *= 263)		
Tested before	629	73%	Tested before	207	79%
First time tester	227	27%	First time tester	56	21%

## Discussion

The RE-AIM framework is a structured approach to evaluate the implementation outcomes of evidence-based interventions. We evaluated the reach and effectiveness outcomes of the I’m Ready program among Black people in Canada and found that the intervention reached Black people with indications for HIV testing, especially those who had never tested before and those who had not tested in the previous 1 year. Moreover, our findings show that less than half of the people who accessed the intervention ordered test kits and reported test results. This is significant because reaching the undiagnosed is central to ending the HIV epidemic in Canada. Moreover, testing is the first step in the HIV prevention and treatment cascade, and it is the first goal of the UNAIDS 95-95-95 targets in which 95% of people living with HIV are expected to be diagnosed and linked to care by close of 2030 (UNAIDS, 2023).

In terms of the “reach” of the program, the intervention reached Black people of different age groups, gender, and sexual identities, and in different provinces and territories. This is noteworthy because, in 2022, 1833 newly diagnosed HIV cases were reported in Canada across all geographic regions (provinces and territories), in different age groups, sex, race, and/or ethnicity (PHAC, 2024). Any testing strategy that can reach a broader target population may increase the likelihood of early diagnosis of HIV, facilitate linkage to treatment, and ultimately ensure good treatment outcomes. In addition, our study found that nearly seven out of every 10 participants reached were first-time testers or had not tested in the previous 1 year. This finding suggests that the “I’m Ready” program may have the potential to increase testing uptake and frequency among Black people with indications for HIV testing who had never tested before or with suboptimal testing frequency. Although our study found that the “I’m Ready” program has the potential to reach this priority population, it is difficult to ascertain which of the features of the “I’m Ready” program may have facilitated the uptake of this intervention among diverse Black people. However, based on research evidence, we know that the use of mobile app technologies has generally been successful in increasing HIV testing coverage and testing behavior among priority populations (Akinola et al., 2021; Bell et al., 2021; Chan et al., 2021; Mancuso et al., 2023). The use of a steering committee for community mobilization and participation could be another reason for the reach of the intervention. The WHO recommends that an effective service delivery model for HIV testing should include three components: mobilizing, testing, and linking (WHO, 2018). Our thinking is that the “I’m Ready” program probably reached diverse Black people in different locations because it has all three essential elements of an effective intervention for HIV testing service delivery. One other plausible explanation is the perceived advantage of using a mobile app for ordering test kits. With the use of a mobile app, an individual is empowered to order HIV self-test kits at their convenience and to the location of their choice, and also perform testing in a discreet, confidential manner thereby eliminating the barriers of inconvenience, cost associated with travel, and fear of stigma often faced by people accessing facility-based testing service. Moreover, according to a report by Media Technology Monitor, 83% of visible minorities (including Black people) own a smartphone versus 75% of all other Canadians (MTM, 2017). In addition, Canadians who identify as visible minorities are 11% more likely to own a smartphone and are more likely to use their phones to access video content online compared to other Canadians. This could be another reason for the reach of the intervention.

In terms of effectiveness of the intervention, less than half of those who accessed the platform ordered test kits. Our findings are different from the outcome reported by Sullivan et al. (2022) in a randomized controlled trial of a mobile app (M-cubed app) designed to increase the uptake of HIVST in three United States cities. In the trial, over half of study participants in the intervention arm ordered HIV self-test kits through a mobile app tool (M-cubed app). Our findings are different from the outcome reported by Tigert et al. (2025) who found that all participants who completed a risk assessment questionnaire ordered test kits through a website (Get-a-kit study). Many factors may influence the uptake of HIV self-test kits with mobile app technology. Mancuso et al. (2023) found an association between ordering a test kit and having a history of HIV testing or intention to be tested in the near future (3 months). There is also evidence to suggest that HIV criminalization policy may be influencing Black people’s willingness to engage and utilize HIV-related services (Patterson et al., 2015). The impact of HIV criminalization policy is more likely to affect Black people who are newcomers, those with precarious immigration status, and those living in shelters; the majority of whom experience structural barriers that may put them at risk of HIV infection (Antabe et al., 2021; Patterson et al., 2015).

Another important effectiveness outcome is the proportion of people who reported their test results. Our findings show that three in 10 persons who ordered test kits reported their test results. Our finding is similar to the outcome reported by O’Byrne et al., who found that only half of Black participants reported their HIV self-testing results (O’Byrne et al., 2025). Although reporting of test results could be used as a proxy for HIV testing, the number of people who tested may be higher than what we found because there are many factors that may influence non-reporting of test results (Antabe et al., 2021; Mancuso et al., 2023; Patterson et al., 2015). Research evidence has shown that concern about the privacy and confidentiality of test results and policy on criminalization of non-disclosure of HIV may affect the way Black people engage and utilize HIV prevention or treatment services (O’Byrne et al., 2025; Patterson et al., 2015; Tigert et al., 2025). For example, Tigert et al. (2025) identified concerns about privacy and confidentiality as the major factors that influenced the reporting of HIVST results on the Get-a-kit website. Further research should explore the facilitators and barriers to reporting of HIV test results through a mobile app, as this may provide useful information to improve reporting of test results and linkage to care. However, it is noteworthy that participants reported both positive and negative results through the platform, highlighting the comfortability of Black people to share the outcome of testing via a mobile platform. This finding may suggest that, in contrast to the framing of their HIV risk, Black people are interested in accessing HIV testing and knowing their HIV status when the testing service is accessible and convenient. The “I’m Ready” intervention may provide expanded access to HIV testing in Black communities in Canada, which can then accelerate early diagnosis and linkage to care.

This study has some limitations. The lack of consensus on how to apply the RE-AIM framework in the evaluation of community-based implementation science research makes it difficult to compare all of our results to other community-based HIV self-testing implementation studies (Gaglio et al., 2013). Secondly, the use of self-report may be a potential source of bias. For example, participants may not accurately remember previous sexual encounters. Lastly, the low proportion of people reporting their test results may also have affected the percentage of positive and negative results reported.

Future research should explore factors influencing the ordering of test kits and the reporting of test results. The limitations of the use of mobile app for accessing HIV self-testing and reporting of test results should be examined using qualitative research methods. Outcomes of this study will provide evidence to improve the effectiveness of the “I’m Ready” program in Black communities.

## Conclusion

The “I’m Ready” intervention is an effective way to reach the undiagnosed in Black communities in Canada. It shows high potential to reach key priority and underserved populations, increase testing (uptake of test kits and reporting of test results), and reach the undiagnosed and link them to care. To maximize the benefits of the “I’m Ready” intervention programs for Black people, barriers to uptake of test kits and reporting of test results should be explored and incorporated into the plan for the scale-up of the intervention.

## Contribution to knowledge

What does this study add to existing knowledge?

- First, our findings will advance the knowledge of strategies to increase the uptake and reporting of HIV self-testing in Canada, particularly in Black communities. Secondly, the use of mobile app and community mobilization as implementation strategies for uptake of HIV self-testing is new. Therefore, our findings on the reach and effectiveness of this intervention will advance the knowledge of how digital technology could help advance public health in Canada.

What are the key implications for public health interventions, practice, or policy?

- The findings from this study will serve as evidence for action for policy makers and public health practitioners, especially for decision-making on whether or not to support the scale up of this intervention. It will also help other practitioners to determine whether to incorporate this intervention into their HIV prevention service model.

## Data Availability

St. Michael’s Hospital is the lead site of the I’m Ready Implementation Science Program, and data collected were stored on servers at St. Michael’s Hospital. Study data may also be transferred outside of Unity Health Toronto and shared with others for purposes related to the conduct of this study and for hosting and/or archiving purposes. Individual-level study data may also be made available to scientific journals, their reviewers, other researchers inside or outside of Unity Health Toronto, or the public.
